# What I Think of Crotalin in the Use of Epilepsy

**Published:** 1915-05

**Authors:** J. Gordon Brydson

**Affiliations:** Bastrop, Texas


### What I Think of Crotalin in the Use of Epilepsy.

BY J. GORDON BRYDSON, BASTROP, TEXAS.

Some time ago I promised to contribute to the "Red Back" a paper dealing with the merits or demerits of crotalin in the treatment of epilepsy. My reason for saying it would be either the merits or demerits was because at that time I was experimenting with crotalin on a few select cases of epilepsy of the so-called petit mal type. And well it was that the subject was left open for either pro or con, for, should I attempt to essay the subject, from my very limited experience, it would be the contraindications of crotalin in the treatment of epilepsy. Not altogether have I based my conclusions upon my own results, but also upon those of Dr. L. B. Bibb, of Austin, through whose kind co-operation another select case of epilepsy was treated with crotalin under different circumstances and surroundings but with the same unsatisfactory results.

Now that in my mind there are no merits to it and that there are reasons for believing that it is contraindicated in the treatment of epilepsy, I offer the following excuse for being so pessimistic:

One very select case I took to my own home and looked after every detail of his daily life, finding it very hard to keep his obstinate bowels open. But by careful diet, special exercise and lots of cathartics this was accomplished. His exercise both mental and physical was regulated, undue excitement was avoided and his surroundings made as pleasant and comfortable as possible. He had not taken any medicine for some months when he had the first dose of crotalin, which was 1-200 grain. This was gradually increased until he was getting 1-75 grain; then for a very short time he seemed to be improving but soon became very much worse. The advise of the people who make, sell and recommend crotalin as a curative for epilepsy was sought, they advised that the dose be reduced, which was done, but with no improvement. All of this time the patient was kept under circumstances that alone, and without any medication, would always improve his condition before. His attacks became more frequent and more severe, then he was sent away to different but almost perfect surroundings, being under the same attendant that he had in my own home and receiving the same dose of crotalin that was recommended, but his condition steadily grew more severe until the crotalin was abandoned and the bromides were recommended. But they were not given at once; however, during this time between the discontinuation of the crotalin and the giving the bromides, the patient became better, then the bromides were resumed and a marked improvement was noticed. This is not said to support the bromides as a treatment for epilepsy and especially not a cure. But of all the medical treatment I believe they are the best we have to relieve the distressed condition of an epileptic when he has frequent and severe fits.

This is certainly no pleasure to me to report the failure of crotalin to affect a cure or even a help to the unfortunate that has epilepsy. There is no disease for which I want to see a specific treatment nor for which I would be more willing to try to help than the epileptic.

Personally I believe that nothing short of some surgical procedure that will facilitate the peristaltic wave or in some other way cause more frequent evacuation of the always obstinately constipated bowel will help the epileptic. I have never seen a case of epilepsy that was not constipated, and they all have great amounts of indican in the urine. Relieving this has improved every one that I have seen except those that were at the same time taking crotalin. Therefore, I believe that crotalin not only fails to act any way favorable, but produces harmful effects upon an epileptic when given.

[Dr. Brydson has written freely and candidly, and we are glad.

Such papers should provoke discussions from those interested. The JOURNAL is open for a discussion on this paper, and we will be pleased to hear from any physician who has had better results from the use of crotalin. We feel sure, too, that Dr. Brydson will be especially pleased to have this matter discussed. Let us hear from you.—ED.]

